# Feline Obesity in Veterinary Medicine: Insights from a Thematic Analysis of Communication in Practice

**DOI:** 10.3389/fvets.2017.00117

**Published:** 2017-07-31

**Authors:** Alexandra M. Phillips, Jason B. Coe, Melanie J. Rock, Cindy L. Adams

**Affiliations:** ^1^University of Calgary, Calgary, AB, Canada; ^2^Department of Population Medicine, Ontario Veterinary College, University of Guelph, Guelph, ON, Canada; ^3^Department of Community Health Science, University of Calgary, Calgary, AB, Canada; ^4^Faculty of Veterinary Medicine, University of Calgary, Calgary, AB, Canada

**Keywords:** feline obesity, communication, thematic analysis, qualitative analysis, diet and nutrition

## Abstract

Feline obesity has become a common disease and important animal welfare issue. Little is known about how, or how often, veterinarians and feline-owning clients are addressing obesity during clinical appointments. The purpose of this qualitative study was to characterize verbal and non-verbal communication between veterinarians and clients regarding feline obesity. The sample consisted of video-recordings of 17 veterinarians during 284 actual appointments in companion animal patients in Eastern Ontario. This audio-visual dataset served to identify 123 feline appointments. Of these, only 25 appointments were identified in which 12 veterinarians and their clients spoke about feline obesity. Thematic analysis of the videos and transcripts revealed inconsistencies in the depth of address of feline obesity and its prevention by participating veterinarians. In particular, in-depth nutritional history taking and clear recommendations of management rarely took place. Veterinarians appeared to attempt to strengthen the veterinary–client relationship and cope with ambiguity in their role managing obesity with humor and by speaking directly to their animal patients. Clients also appeared to use humor to deal with discomfort surrounding the topic. Our findings have implications for communication skills training within veterinary curricula and professional development among practicing veterinarians. As obesity is complex and potentially sensitive subject matter, we suggest a need for veterinarians to have further intentionality and training toward in-depth nutritional history gathering and information sharing while navigating obesity management discussions to more completely address client perspective and patient needs.

## Introduction

Obesity affects approximately 11.5–63% of the canine and feline population in industrialized nations (1–7). Obesity is both a medical issue and an animal welfare concern that can cause or expedite a multitude of health problems in dogs and cats (8). Potential sequelae in dogs and cats include diabetes mellitus, osteoarthritis, cardiovascular changes, and decreased lifespan (9). In North America, obesity rates of pets mirror those of the human population (10). Obesity is considered difficult to discuss, and human physicians often struggle to find consistent and clear recommendations around management (11). In a study examining western media, Degeling and Rock (12) report that companion animal obesity and diet are complex and sensitive issues. Companion animal obesity could be addressed with specific skills that effectively facilitate a discussion regarding weight loss and diet with intentionality and targeted approach toward clear and sensitive discussions around obesity management. As obesity is, therefore, a nuanced phenomenon among people and their pets, veterinarians are faced with a complicated health issue to discuss and manage with pet owners. Kurtz and Adams (13) suggest that veterinarians face struggles when broaching sensitive subject matter within cross-cultural contexts, and point out the need for an increased emphasis on certain professional communication skills by the veterinarian in order to more effectively meet the needs of clients and patients. It is possible that communication that has to do with weight and obesity also requires an emphasis on select communication skills to be able to work effectively with clients and their pets.

Despite the lack of research in this area, the importance of nutritional counseling in veterinary settings has been acknowledged by the veterinary profession to be important for the veterinary industry (14–16). In 2010, the American Animal Hospital Association identified nutrition as the fifth vital assessment that should be included in every companion animal health care exam. Incorporating nutritional assessment into regular patient care is critical for maintenance of health and prevention of disease and injury (15, 16). The specific formulation of questions asked by the veterinarian can influence the precision and comprehensiveness of the nutritional history taking (17). Furthermore, German (18) suggested that incorporation of nutritional assessment and recommendations regarding the care of small animals helps to develop a partnership between the owner and veterinary healthcare team, resulting in healthier pets.

In veterinary medicine, dietary management is considered the keystone approach to weight management for cats and dogs, generally through creating negative caloric imbalance, potentially aided by the use of any number of specialized diets (18). However, poor compliance by pet owners through overfeeding has been consistently identified as the most likely reason for poor weight loss outcomes, which can lead to owner and veterinarian frustration and failure of treatment (8, 19). Complicating the nature of obesity and weight loss approaches in companion animals is the perspective that health is not maintained if a pet is overweight, despite not enough empirical evidence investigating parameters of health in overweight pets, and how overweight pets might still live a healthy lifestyle.

There appears to be disparity between the viewpoints of veterinarian and client viewpoint. Many owners normalize their dog’s weight and minimize the severity of the problem, even when deemed as overweight by a veterinarian (20). Bland et al. (21) found that 97% of veterinarians reported pet obesity as an owner-driven issue, complicating the nature of discussions at the veterinary office. Furthermore, in a survey of 1,104 dog or cat owners, 32% (*n* = 356) of pet owners reported having an overweight or obese dog or cat while only 0.3% (*n* = 3) perceived obesity as a health concern (22). The bulk of companion animal obesity research has examined dogs despite cat ownership outnumbering dog ownership in North America. Although there are biological and social differences in feline versus canine health management, the canine-based research into obesity management can be used to inform the feline-based research in this area, as there are many parallels in how the average cat and dog owner manage their pets, and some generalizations in approach can be drawn.

Cat ownership exceeds dog ownership in Canada and the United States (23, 24). However, cats tend to receive less veterinary care in comparison to the care that dogs receive, even if they live within the same household (25). It follows that feline care is an area of growing interest in veterinary medicine (26). In the United States, 52% of cats had not been to the veterinarian in at least a year and only 37% of cat owners indicated that they go to the veterinarian for routine wellness examinations (27). As such, there are further opportunities for veterinarians to collaborate with cat owners to prevent or manage companion animal obesity and related-health complications by increasing routine wellness examination compliance by improving communication with clients and providing obesity management recommendations and explanations (25).

To date, there is minimal research pertaining to veterinarian–client–patient communication regarding companion animal obesity and diet. The value of effective communication within the veterinarian–client–patient relationship is well discussed within the veterinary literature (28). Improved uptake and adherence to veterinary recommendations by clients has been shown to be linked to veterinarians providing relationship-centered care and concise recommendations (29). Effective recognition, intervention, and management of obesity by the veterinarian are important when considering the multitude of health concerns that are influenced or complicated by obesity (8). Therefore, it is likely that effective communication plays an important role in achieving favorable health outcomes in relation to obesity management. Furthering our understanding of veterinarian–client communication in relation to obesity management of companion animals is an important step in developing communication strategies for veterinarians to manage companion animal obesity. Our aim in this study was to further characterize and understand communication practices between veterinarians and clients in observed veterinary appointments and specifically to explore the discussions around obesity management taken from a sample of feline primary care consultations.

## Materials and Methods

This study protocol was reviewed and approved by the University of Guelph and University of Calgary Research Ethics Boards.

### Sample

Data were drawn from a pre-existing archive of 365 English-speaking video-recorded veterinarian–client–feline patient interactions collected in 2006 from small-companion-animal veterinary practices in South-Eastern Ontario (30). Of the total 365 visits collected, 284 clients consented for their appointment to be archived and utilized for secondary analysis of veterinarian–client–patient interactions (30).

**Figure 1:**
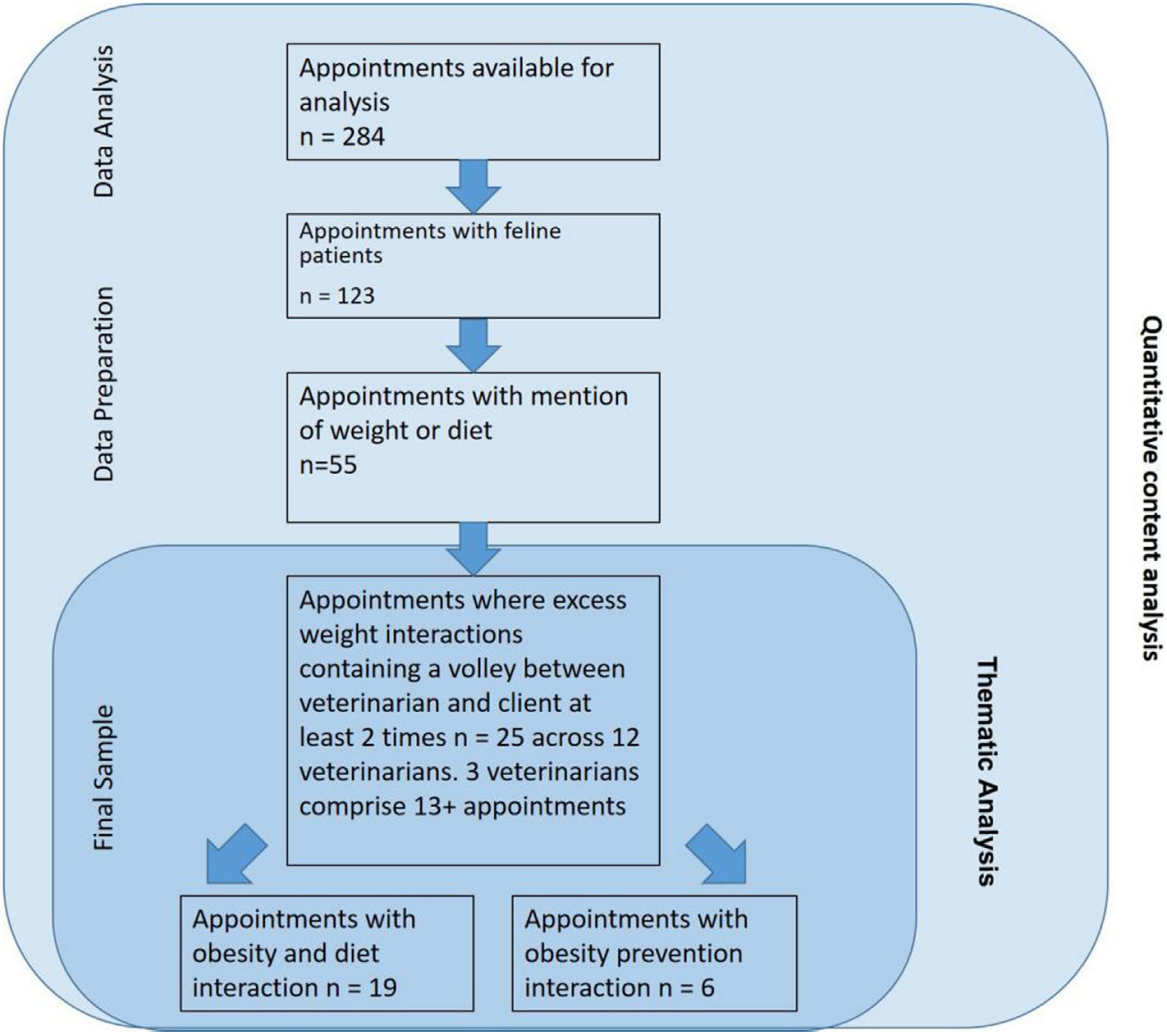
Flow diagram of thematic analysis to examine feline obesity talk between veterinarians and clients.

The archive of 284 videotaped appointments was screened for feline patients (Figure 1). One hundred and twenty-three (43%) appointments were found to involve feline patients and these appointments were further screened for discussion of weight and diet. Next, the context notes and videos were used to identify interactions in which the discussion of excess weight occurred. Discussion was defined as a verbal exchange between veterinarian and client(s) about excess weight and diet that went back and forth between the veterinarian and client at least two times during the visit. Weight-related visits that were excluded from the analysis were:

- Weight and diet discussions that did not volley between veterinarian and client at least twice.
- Feline patients that were seen for unexplained weight loss.
- Feline patients not verbally assessed by veterinarian or client as overweight, obese, having had an excessive weight gain or any other colloquial term referring to increased body weight with the exception of visits where the veterinarian offered preventative advice against further weight gain.

Appointments where obesity prevention discussion occurred between veterinarian and client were included in our sample. In these cases, the patient was identified by the veterinarian as borderline obese or the need to prevent further weight gain occurred. Upon preliminary analysis, the researchers elected to incorporate this subset in analysis, as the discussions revealed congruent themes as excess weight visits.

### Analysis

Partial transcripts had been transcribed *verbatim* for previous analysis by Hannah Wheat and Clare MacMartin (17), and additional transcription of visits containing weight and diet talk that met selection criteria was provided by the primary author. Our interpretivist approach was influenced by the standards of communication set in the Calgary-Cambridge Guides (CCG) (31, 32). The CCG is a widely used instrument for teaching and learning clinical communication skills across veterinary and medical curricula [(31), Table 1]. Following screening of videotaped appointments and familiarity with data sets, videotaped appointments of feline obesity discussions were transcribed using Transana™ software. Transcripts and video footage were studied and additional non-verbal information (annotations) and context notes were described (33).

**Table 1:** Calgary-Cambridge Guide basic framework (31).

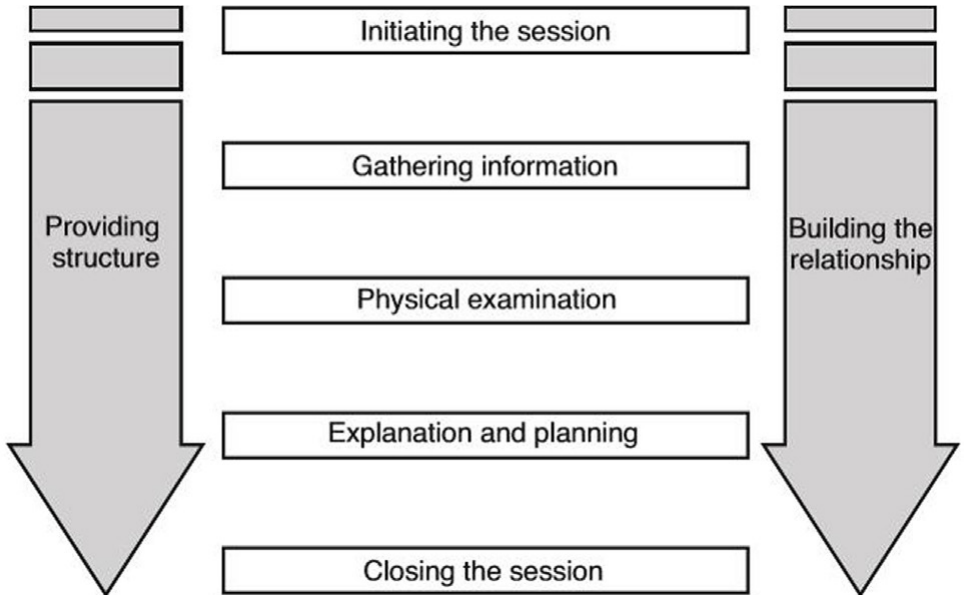

Thematic analysis (33), an interpretivist and inductive qualitative analytical strategy was used to examine the discussion sequences, details, and underlying themes of veterinarian and client discussion of pet obesity. The interpretive analysis of the first transcript and initial notes formed the basis of an evolving and iterative coding system, organizing data into meaningful groups, and provided direction for further exploration of transcripts to follow. The transcripts were read and coded by Alexandra M. Phillips. The data set was worked through systematically to identify new or re-occurring codes during interactions between veterinarians and clients within the context of the research objectives pertaining to weight and diet discussions. Persistent codes and relationship between themes and subthemes, behaviors, and responses as well as anomalous events were identified for participating veterinarians and clients. Measures to enhance the rigor of the thematic analysis and in turn the trustworthiness of the findings included keeping an audit trail of transcripts and codes, code definition decisions, and memos describing the analytic process (34, 35). As new themes emerged, transcripts were re-assessed and re-coded until no new themes emerged such that data saturation was reached (33). Coding of transcripts was managed by Nvivo™ software. Transcripts were corroborated with video footage and when necessary, notes were made about the obesity status of a patient when pertinent to highlight the communication. Alexandra M. Phillips has been trained by veterinarians to assess feline body weight. For quality assurance, the coding structure and differences in interpretations were reviewed, discussed, and resolved to consensus (34) by the authors. Areas of uncertainty or disagreement were resolved to ensure that themes were consistent (35) and were reviewed by the authors.

## Results

### Sample

Application of the preliminary inclusion and exclusion criteria resulted in 25 feline obesity and diet discussions from the original sample of 123 interactions involving a feline patient. Nineteen of these visits were found to contain excess weight and diet discussion, and six were identified to contain excess weight *prevention* discussion by the veterinarian to counsel against excess weight gain in pets that were not currently assessed as obese (Figure 1).

There were 12 veterinarians involved in the 25 appointments, which comprised 34 clients and 35 patients. Nine of 12 (75%) veterinarians and 18 of 25 (72%) clients were female. Veterinarians were aged 26–51 years (mean = 36.5; median = 32). Clients were aged 23–77 years (mean = 44.2; median = 42). The mean number of years in practice for veterinarians was 9. In 22 of the 25 visits (88%), the client had a previous relationship with the veterinarian that conducted the examination. Most (80%) of weight and diet discussions were initiated by the veterinarian. The majority of obesity dialog and obesity prevention talk (80%) occurred during routine wellness exams rather than exams where the patient was being evaluated for a specific health concern.

Over half of the excess weight discussions in the sample were generated from a few individual veterinarians (one veterinarian contributed 28% and two others contributed 12% each of the interactions studied). Within the entire data archive of veterinarian–client–patient interactions from which the sample was drawn, 5 of the 17 veterinarians had no appointments that fit the inclusion criteria. Many veterinarians had very few visits that met inclusion criteria from the archived visits (range = 7 visits; mean = 2.1 visits; median = 1.5 visits). With the exception of one visit where the patient was in for a follow-up weigh-in, client reasons for bringing feline patients in for veterinary assessment were not due to obesity. Our analysis captured a diversity of communication events surrounding weight and weight-related diet discussions. Obesity discussions varied widely in terms of their complexity, duration, and depth by the veterinarian and/or client, ranging from brief and vague statements to detailed discussions between veterinarian and client.

### Themes

**Figure 2:**
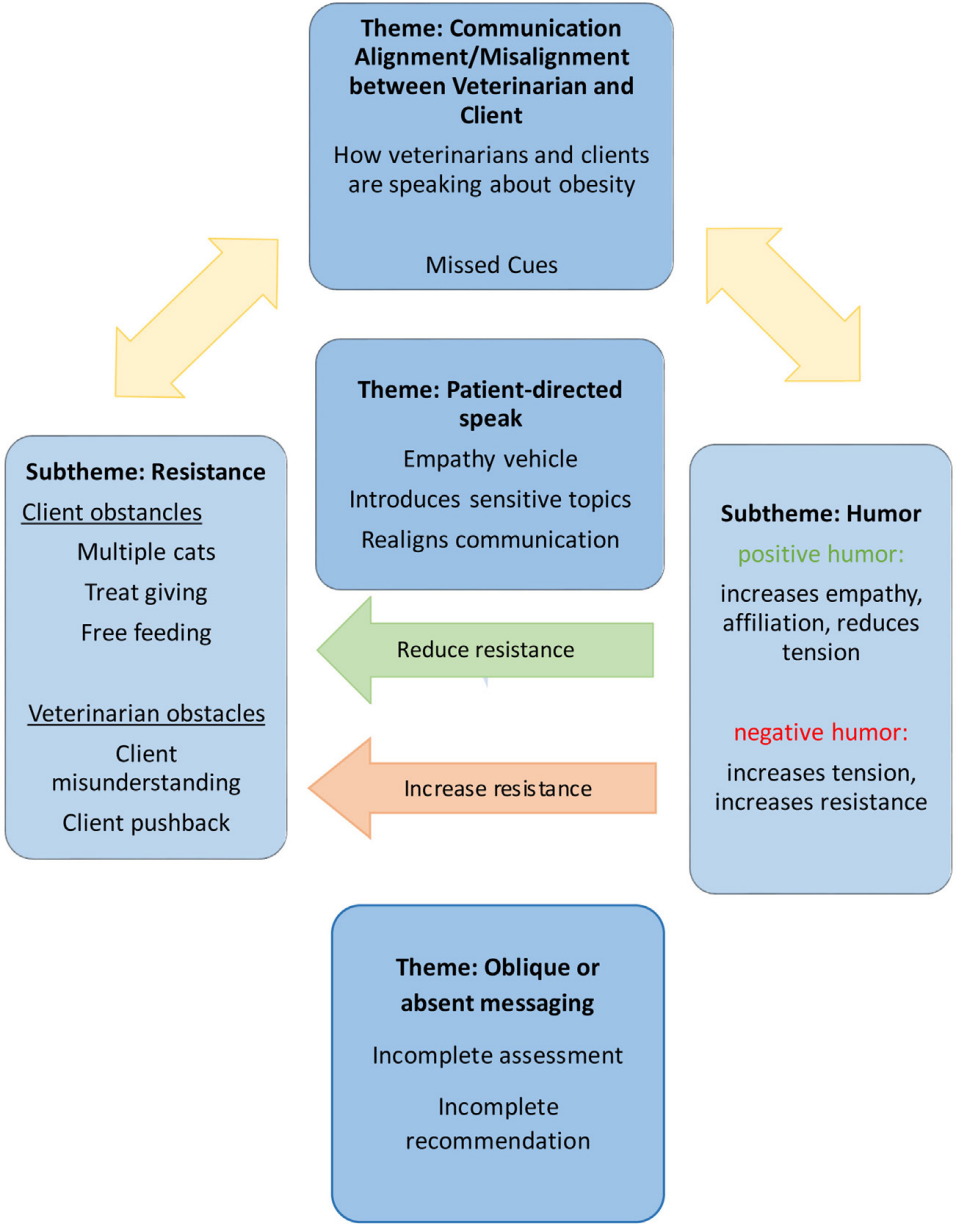
The themes and subthemes in feline obesity communication from thematic analysis of feline–veterinarian–client consultations and the relationships between them.

Themes central to obesity and obesity-related diet communication were related to the alignment or lack thereof (misalignment) of communication between veterinarian and client. Alignment refers to the veterinarian and client conversation seeming to flow with all parties communicating openly and effectively and utilizing communication skills as outlined in the CCG. Misalignment refers to the veterinarian and client conversation differing in terms of communication topic, understanding, intent, or goal. This presented in a number of ways, most commonly by the veterinarian or client asking a question and the other responding in a manner that does not directly answer the question, or when the veterinarian or client failed to respond to verbal or non-verbal cues or queries about weight and diet. A discussion was determined to be misaligned if weight and diet discussion topics were ignored, dropped, and misunderstood (such as if a question was asked and answered with information that does not address the original question), or the veterinarian and client otherwise did not appear to be communicating fully about weight and diet (such as if questions by one party were left unanswered). Themes that emerged in relation to alignment or misalignment were client/veterinarian resistance, oblique communication from veterinarian to client, humor, and patient-directed speak. Themes and subthemes were interconnected, with numerous components influencing and interacting with other themes (Figure 2). For example, patient-directed speak (theme 2) was often framed in a humorous manner (subtheme 1.2) and served to build the relationship and reduce communicative resistance (subtheme 1.1), demonstrating how patient-directed speak and humor themes interact with communicative resistance themes.

#### Theme 1: Communicative Alignment and Misalignment between Veterinarian and Client

Veterinarians and clients were often misaligned in their discussion of weight and diet. A common manifestation of misalignment was how veterinarians and clients often communicated with different descriptors when discussing diet. Veterinarians tended to focus on brand, quantity, and perceived quality of diet. When a veterinarian recommended a change in cat food, they primarily focused on diets marketed to prevent dental disease. Clients tended to focus on brand name, shape, and flavor or type of ingredients. These differences in terms underscored the difference in the way that veterinarians and clients talked about food. In one visit, the veterinarian recommended a decrease in amount of food, and asked the clients what kind of food they were feeding their cat. The clients did not know what “kind” of cat food they feed and describe it as “square” and “colorful.” When the veterinarian queried whether it was a weight loss or lower-calorie formula one of the clients respond with “just cat food.” After the veterinarian told the clients that most foods come in a low-calorie formula, the weight and diet topic was abandoned by the veterinarian and clients for the duration of the appointment. The veterinarian does not take any further nutritional history or make any further recommendations nor offer to help navigate management strategies for weight loss. This exemplifies a misalignment between veterinarian and client in terms of how they identify diet and the unfinished recommendation process from the veterinarian.

In a different visit, another veterinarian queried the client about the food that she feeds her two young cats. The client could not identify the food by brand, formula, life stage, or caloric content, but she could describe it as “organic” and “natural” and “from the pet store.” The veterinarian revealed that one of the cats was becoming “pouchy,” and asked the owner if the cat is free fed or given measured amounts. The owner (who has a third cat that was absent) said the cats are given measured amounts but went on to describe providing food in a single bowl that all cats share (and, therefore, does not measure individual intake). The client asked the veterinarian if the organic food she feeds is too high in fat, and the veterinarian responded by saying that an adult food “is fine,” and therefore does not clearly answer the question.

ID: 12033211
Veterinarian (V): Yeah you wouldn’t want him [to get any bigger] no,
maybe he’s getting more [food].
Just cut back otherwise.
Client (C): A different kind of food maybe?
V: Yeah it might be too…
C: Maybe there’s too much fat in the organic food?
V: I don’t know the content but…Certainly an adult food I was gonna
say is fine because of their size.

At this time the veterinarian abandoned weight, fat content, and diet discussion, but the client persisted with further questions regarding a diet change to curb weight gain.

V: Very nice (*fur*) eh? I was just going to say. Must have…must have lots of fat in that (*laughs*) food.
C: Maybe I should change it to (*laugh*) something else. A light version of it or something.
V: You can always bring in a label we can have a look cuz I don’t know there are so many different, comparing them is, uhh.

The veterinarian did not further develop this client’s thoughts on cat food selection. After some discussion around the cat’s weight between the last visit and the present, the client questioned the veterinarian for the third time about the patient’s diet and whether she might change it to mitigate weight gain.

C: So, I’ll see if this organic food…maybe it has…
…maybe the organic food has a like a lighter version of it (*veterinarian nods*) or I’ll check that size.
V: (*looking at client*) Has your other cat been on the same food for…
C: Yes
V: A long time? Yes? As well.

After this, the veterinarian commented on the second patient’s size, although she did not follow through with a clear verbal assessment of the patient’s weight: the veterinarian did comment that the cat was “a little bit bigger, but she’s got really long fur, so she looks bigger I think than she is. They’re probably close to the same size…” No follow up with weight management occurred for the remainder of the visit. The client had introduced the appropriateness of her food choice for the cats in terms of weight management numerous times throughout the interview to elicit veterinarian assessment on the diet and health of the cats.

During the final moments of the appointment, the veterinarian prepared to remove one patient from the room for a procedure. Despite the assessment that the cats need to “cut back” on dietary intake, the veterinarian gave the client a few treats to give the remaining patient even though the veterinarian said that she is not “supposed to have treats,” alluding to her weight. Before leaving, the veterinarian supplied a small number of treats to give to the patient, and placed the remaining supply in a drawer away from the client. While the veterinarian was out of the room, the client gave the cat all the supplied treats, as well as took many more from the veterinarian’s supply to give to the cat, contrary to the client’s discussion of a possible diet change to curb weight gain. This interaction serves as an exemplar of the misalignments and mixed messaging between veterinarian and client during weight and diet discussion. It is unknown if client’s excessive treat giving is indicative of not understanding the veterinarian’s recommendation to minimize treats and excess food; understanding the veterinarian’s recommendation but not being clear on the impact of excessive treating and food; or the client understanding and recognizing the impact of her actions, but deciding to give excess treats anyway.

The veterinarian spoke about weight management in terms of reducing the ration size and the client spoke about weight management in terms of a change in diet. As was typical for the data, the veterinarian provided unclear assessments and vague recommendations of obesity management and demonstrated limited response to verbal cues from the client. Commonly in this investigation were instances where the veterinarian or client would ask a question and the respondent did not directly or accurately answer.

##### Subtheme 1.1: Communicative Alignment/Misalignment: Resistance

Resistance is defined as a barrier or point of difference in communication, where typically a rebuttal, objection or deferral, or subject change was offered in response to misalignment. Typically, resistance occurred if the veterinarian had been making an assessment or recommendation that the client did not agree with due to foreseen obstacles at home for weight management. Veterinarians also typically seemed to meet greater resistance when pursuing a topic singularly, rather than circling back to the subject. Resistance was often an outcome that emerged when communication asymmetry occurred and attempts to realign failed or did not occur. Common sources of client resistance to weight management in many visits included: feeding of multiple cats in a single household and the difficulty in addressing the individual dietary needs of pets; supplementary feeding, such as treat giving; food palatability; and free feeding versus measured amounts. Veterinarians sometimes changed tactics when met with resistance, which often resulted in partially or fully abandoning their recommendations, or dropping the topic until later in the visit.

There were several instances when the veterinarian was not in the exam room and the clients talked among themselves, offering a candid view of their thoughts and perspectives. In one visit, the clients were discussing the patient’s weight and acknowledged that the cat was likely to be assessed as overweight and discussed their unwillingness to address the concern further before the veterinarian entered the exam room. It is unknown if the patient’s weight had been addressed at a previous visit, though a previous difference in opinion between veterinarian and client might have caused the clients to prepare themselves and their position. When the veterinarian was assessing the cat, the clients volunteered their resistance and reluctance to mitigate any changes in their management of the cat’s weight. This client pair had identified a challenge of adhering to the veterinarian’s recommendation because they have more than one cat to feed. The veterinarian did not meet the client’s resistance with complete abandonment of the recommendation but reflected his understanding regarding how challenging managing weight in a multiple cat home by saying “yeah its nuts, I know” in a kind and gentle manner after the client expressed difficulty implementing the recommendation. “Yeah its nuts, I know” had an affiliative and empathetic quality in response to the client’s home situation. Later in the interaction, the veterinarian offered some creative solutions for the clients based on their specific challenges that appeared to have generated interest from the clients. By the end of the appointment, the clients appeared to have taken up the veterinarian’s suggestions by agreeing to look into some of his suggestions. The reader may notice that humor is a theme that appears in this interaction as well.

##### Subtheme 1.2: Communicative Alignment/Misalignment: Humor

Humor was a common tool used to realign communication or reinforce the relationship between veterinarian and client by attempting to further build rapport during obesity discussions. Humor was an element utilized by both client and veterinarian in many visits involving the discussion of excess weight of a feline patient. Humor frequently appeared to be well received by the other party, where it would generate laughter, affability and appear to lighten the mood, or otherwise create an environment that invited further expansion on the discussion point. Humor also appeared to lessen tension or attempt to lessen tension surrounding weight and diet discussion, while still drawing attention to the topic. Veterinarians frequently utilized humor to deliver news regarding the patient’s overweight status, or when delivering recommendations pertaining to weight and diet. In the following example, the veterinarian revisited the subject of a dental diet recommendation that was made at a previous visit for a client with two cats, one of which was overweight. The first patient had been identified by the veterinarian as a “big boy,” and the veterinarian and client had a light-hearted discussion about his body condition. However, the owner alluded to the second patient “Peanut” remaining overweight and the veterinarian responded to her previous diet recommendation follow-up with humor:

ID: 01010311
C: I did give it to him but he um,
V: He didn’t like it?
C: He didn’t really like it much but like
(*looks up but not at anyone*)
C: His sister, Peanut…
V: Loved it too much?
(*both laugh*)

This affable joking seemed to facilitate further humor and open discussion regarding the body condition of both patients. A detailed discussion between the veterinarian and client about body condition assessment and feeding ideas followed. The client also elaborated on information regarding the feeding and exercise activities within the patient’s lives.

Within the studied interactions, humor was frequently used as a communication tactic to attempt to diffuse tension between veterinarian and client. This was commonly well received by the other participant, but in some instances attempts at humor remained unreciprocated, or negatively influenced the encounter. In these instances, an attempt at humor by either the client or veterinarian was ignored or resisted by the other party. In the following example, the veterinarian was seeing a patient with an established history at the practice. The veterinarian appeared affable (smiling, gregarious) and began the discussion of excess weight in relation to the client’s cat by undermining his colleagues’ previous overweight assessment, implying his colleague was wrong about the obesity assessment, although confirming the cat was overweight. The client had been discussing the changes she had made to the cat’s feeding management based on the previous visit with the colleague veterinarian and stopped sharing this information when the current veterinarian undermined the assessment using humor. The veterinarian contradicted himself by agreeing that the cat was overweight, followed by further undermining his own and his colleague’s weight assessment by saying, “It’s fine,” referring to the cat’s weight. Tension appeared to build between the veterinarian and client based on the owner’s non-verbal signals and withdrawal from the discussion. As the client’s involvement in the discussion decreased, and the veterinarian’s speech and body language appeared to reflect a lack of awareness about the owner’s change of demeanor, in that he continued to use similar humor tactics despite previous attempts had failed to illicit response. The veterinarian continued with his physical exam and directed his humor directly toward the patient. He joked that “nobody likes you” to the feline patient, due to his weight. No reciprocation of humor occurred at any point from the client:

ID: 04001311
V: Yes. We’re just-ah as long as we’re all aware
that she’s on the heavy side
C: Yeah.
V: Instead of the light side.
C: Oh yeah.
V: That’s okay. That’s fine by me.
Oh now if-if you keep purring I can’t hear
your heart. You know that eh? C’mere
(*trying to auscultate heart, looking into cats face*)
PShh
Nobody likes you
You’re fat
I didn’t mean it
I didn’t mean it (*finishes auscultation*)

In this case, the veterinarian utilized somewhat patient-effacing humor to deliver his assessment of the patient’s weight status. While the client engaged less and less during the interaction, the veterinarian continued with humor attempts that appeared to not contribute to the relationship, if not detract from it. The weight topic was dropped and no further weight or diet discussion occurred.

More commonly within the studied interactions, humor use seemed to be a means in which the relationship among the veterinarian and client was strengthened, and a conduit for the expression of empathy. Generally, affiliation was built alongside tension-diffusing humor between veterinarian and client. As an example, one veterinarian used humor during a feeding strategy recommendation to add dynamism to her explanation:

ID: 11012811
V: Yeah. Just like when we eat right?
C: Yeah yeah yeah.
V: It’s like they are people right? Ah, like they recommend you to eat like you know several times a day not as in all day long right? So it’s the same. Because cats don’t know how much is too much.
(*everybody laughs*)

The humor seemed to refresh the discussion and invited further discourse over this specific patient’s feeding habits and was followed by the client asking the veterinarian to provide an opinion about the cat’s weight. The veterinarian had appeared to gain rapport with the clients as demonstrated by the client’s friendliness and willingness to share information. Humor enabled the veterinarian to circle back to more in-depth discussion on the patient’s feeding habits. The client was very receptive to the veterinarian’s recommendations: “yeah, whatever you recommend.” Later in this same visit, the owner shared an earnest account of her overweight cat’s activities:

C: She’s full, she’s solid. She’s is solid.
V: mmhm
C: She’s solid muscle.
V: mmhmm
C: They play like, all night.
V: mmhmm
C: If you were standing outside my house you’d swear
I have two Clydesdales running up and down the stairs (*tech laughs*) but she does hang, Hehehe (*C gestures belly fat, laughs*)
V: yep. And that’s where they put their extra fat first
C: and she is chubby. oh
V: it looks like a lose skin flap around their tummy swaying back and forth.

Clients frequently used humor when acknowledging the veterinarian’s viewpoint and sharing their animal’s history. In the case above, humor was also a vehicle for the owner to acknowledge her cat’s weight with humor and for the veterinarian to provide further information regarding her assessment of the cat’s weight to the client.

Typically, when a client was resisting an overweight assessment, an explanation utilizing humor seemed to be effective in aligning the communication between veterinarian and client, diffuse tension, and convey empathy. This was often in the form of a gentle joke, patient-directed speak (see below), and so forth. Affiliative humor was not exclusively used to diffuse tension; as in many cases, humor appeared to be used to cement an already affiliative relationship between the client and veterinarian.

ID: 12032511
V: (*Examining P*)
C: Unfortunately (client’s husband) has been sort of free feeding them
like when they meow, he feeds.
V: MmmHm (*Nodding*)
C: So I think I’m feeding the right amount but then he sneaks in.
V: He sneaks in the food?
C: Yeah, I actually caught him in act
(*laughing*) the other day, (*laughing*)
V: It’s always the husband,
C: Yup.
V: Hahaha that’s it.

Humor and laughter were often used by the veterinarian or client in conjunction with reporting an increase of weight. There were multiple instances of clients and/or veterinarian laughing and joking at the weigh-in event when an increase in weight was reported. Clients also frequently used humor as a means of introducing the topic and their awareness of their cat’s weight, sometimes as a pre-emptive conversation point before the veterinarian brought up the weight topic.

ID: 40023811
V: So how are the girls doing?
C: they’re good. Stella’s um
V: excellent
C: nice and…chunky?
V: Yes (*smiles, looks down at notes*)
(*C chuckles*)

Words like “porky,” “chunky,” and “pouchy” and other colloquial terms for referring to their pet’s weight were used by both veterinarian and client, which largely appeared to result in laughter, smiling and affability from the other party. Alternatively, when a client was seen to be resisting or minimizing the veterinarian’s excess weight assessment for a feline patient (such as denial, discussing obstacles for treatment, reluctance, and so forth), veterinarians often used humor to soften the observed or anticipated resistance by the client. This resulted in the client continuing the conversation about the cat’s weight, or alternatively and less frequently, the client was minimally responsive and the topic was dropped.

#### Theme 2: Speaking to/for the Patient (Patient-Directed Speak)

Both veterinarians and clients spoke to and ventriloquized the patient, i.e., spoke for the patient. Clients and veterinarians spoke through or to the patient when delivering weight status, at the weigh in, or discussing management. While patient-directed speak was frequently utilized in conjunction with humor, this was not exclusively the case. Patient-directed speak appeared to be used to realign the conversation between the veterinarian and client and was typically seen as a tool to project humor; to empathize with the patient and the veterinarian or client; or to convey messages. Generally, veterinarians utilized patient-directed speak throughout the weigh-in and physical exam, and in other delicate moments, including re-addressing weight management strategies and managing the patients when they became upset during the physical exam.

In the following example, the veterinarian utilized patient-directed speak to acknowledge the patient’s improved status (the cat is an adult who was rescued at a young age) since her last visit and to discuss the cat’s weight gain:

ID: 17018911
V: oh my goodness look how good you look now you are just gorgeous.
You’ve come a long way since then. It’s the early days, huh?
C: Mmhmm
V: It’s okay. The scale is a fine place to be isn’t it. 5.37.
Just a little bit of weight gain there.

The clients were witness to this interaction between the veterinarian and the patient and they heard the veterinarian’s viewpoint through the communication to the patient. The veterinarian built affiliation between herself and the client by praising the cat (and, therefore, the owner) and gently introducing the increase of body weight, which is circled back to and developed further later in the visit.

The following interaction shows two clients waiting for their appointment prior to the veterinarian’s arrival. The clients are conversing about the weight of their cats and then resistance to make changes to their current feeding practice.

ID: 40023811
C1: (*To cat*) I think we are going to have a little discussion about your weight, probably.
And guess what? We’re probably not going to do anything about it
C2: (*to C1*)
she turned around when you said that hehe
C1: She says okay, if you never do anything about that, I’ll come back.
C2: (*chuckles*)

Later during the examination when the veterinarian is present, the veterinarian used patient-directed speak (and humor) to introduce the topic of weight and introduce his overweight assessment. The client resisted the veterinarian’s attempt to talk about the cat’s weight and the veterinarian used patient-directed speak again to ventriloquize:

V: (*To patient 1, after small talk with clients*)
Hi. How are you? Yes
You’re not missing too many meals are you?
C1: Hehehe
V: And there ya go slim (*to patient 2*)
V: How are you? (*to patient 2*) Hehehe
How you doing?
C1: And the weight thing is not (*hand waving, dismissive form*) going to be adjusted cuz there’s too many animals to feed.
V: (*softly*) Yeah. Yeah probably not I know
C2: Well the thing is
we don’t even give her that much
I don’t know where she gets all this food
V: (*still kneeling, looking at cats*) She says I just don’t burn it off well.
(*to cat*) Huh, hey you? Belly girl.

Commonly in our data, we found that the veterinarian used patient-directed speak that enabled them to gently reintroduce a sensitive topic, and provide assessment and perspective on the cat’s weight. Using these strategies, the veterinarian was able to circle back to the weight and diet topic several times, and identify and dispel some of the client’s reasons for resistance. This helical strategy seemed to encounter less resistance from clients and appeared to be a vehicle to offer pre-diagnostic commentary as a less-formalized means of introducing a topic, and preceding a formal diagnosis (36).

#### Theme 3: Oblique or Absent Messaging

Typically, among the visits included in this study, the veterinarian did not discuss weight beyond the initial report of weight increase nor offer recommendations on weight or diet management. In several of the visits, the patients appeared to be obese from review of the video-recorded interaction per 5-point body condition scoring (37). In one example, a veterinarian and a client briefly discussed the cat’s weight of 9.8 kg. At the end of the visit, the veterinarian said that the cat was gradually losing weight although the client and medical record indicated the patient’s weight was similar to the previous year. No further discussion regarding weight occurred. The client normalized his size and the veterinarian abandoned the topic.

Our investigation indicated multiple visits where the veterinarian did not give a clear assessment of the patient’s overweight status until pressed by the owner. If the owner had not probed, a clear assessment may not have taken place. In these cases, the weight discussion typically ended after the assessment without further recommendations or strategies for weight loss discussed by either party.

When discussing weight loss, the veterinarian’s recommendations typically consisted of cutting back on quantity of food, and/or looking for a lighter version of their current diet. Discussion regarding exercise and activity was virtually absent, with the exception of two interactions where the patients were outdoor cats. With one patient, the veterinarian told the client that the cat would likely lose weight once the weather warmed and the cat could spend more time outside. The veterinarian implied that increased time outdoors would lead to weight loss, although no true weight loss or obesity management planning occurred.

Prevention-specific obesity discussions occurred in six visits across three veterinarians. These discussions took place with the owners of non- and mildly overweight cats. These discussions mirrored the discussions that the veterinarians and clients had when the patient was obese in the structure and themes seen, although veterinarians also occasionally discussed the importance of obesity prevention as a means to prevent chronic illnesses related to obesity. When discussing weight assessment and recommendations, veterinarians were typically oblique. Preventative talk usually pertained to the patient’s current weight, which was either still currently ideal or near-ideal, or borderline overweight, followed by the veterinarian warning against further gain. This often precipitated a conversation about the client’s current feeding strategies although fell short of discussing concrete strategies to prevent or manage further weight gain.

## Discussion

While feline obesity is a growing concern (9), the current study showed that there may be several missed opportunities for weight and diet conversations during veterinarian–client–feline patient interactions. Given that approximately 50% of cats in North America are overweight and obese (38), we could expect to have seen our data set has two to three times more the number of excess weight discussions. Considering the size of our final sample and additional number of patients that appeared overweight according to BCS on video footage, this suggests participating veterinarians did not actively broach the weight topic with owners of overweight patients. In addition, the overlapping and interconnected nature of the themes revealed in our investigation highlight the complexities of obesity discussions for veterinarians and clients, as a single topic might influence and be influenced by multiple themes.

### Communicative Misalignment between Veterinarians and Clients

Fundamentally, veterinarian and client communications reflected the differing perspectives, beliefs, and values held by both parties. Communicative misalignment between veterinarian and client was a regular theme within veterinary visits investigated in the current study. Misalignment between veterinarian and owners has previously been described with reference to perceptions of pet obesity (20). According to survey of dog owners where the dogs were overweight, 39% (*n* = 48) of owners thought their pet was at an “acceptable” weight despite the overweight assessment by the veterinarian. This misalignment is mirrored in other veterinary studies examining owner perception asymmetry with pet body condition score (39), further in pediatric literature regarding overweight children–parent–physician triads (40). With respect to dogs, previous studies have reported misalignment between veterinarian and client in regard to obesity management strategy preference (41). Our findings support this, as clients identified obstacles for simple obesity management techniques such as measuring and rationing intake. This might provide more clarity on the lack of owner compliance in dietary and activity intervention measures set forth by veterinarians. In a study characterizing the communication between veterinarians and standardized clients during discussions regarding end of life, misalignment existed between veterinarian and client responses regarding the quality of the interaction. Veterinarians rated the quality of the experience to be much better than the client’s perception of the visit. For example, clients perceived that veterinarians failed to explore how manageable treatments would be 77% of the time, whereas veterinarians responded this way 17% of the time (42). Shaw et al. (43) reported that the least amount of conversation during the veterinary consultation was devoted to gathering information (9%). As such, it might be difficult to ascertain a clear idea of client perspective without enough information gathered. Gaps in information might impede formation of an effective management plan with the client and outcomes of care for the patient might be impacted (31). Weak information-gathering might account for the inconsistent address of weight management for patients in this study, as the explanation and planning of weight loss strategies would be dependent on the client/patient needs and perspectives gathered by the veterinarian (31).

By letting conversation topics drop off before a clear assessment or management discussion occurs, opportunities for improved veterinarian–client relationship building and weight management were missed. Dropping a topic may serve to allay tension or guilt when sharing or receiving sensitive subject matter, but this may have undermined the assessment and created ambiguity for clients. As the majority of weight and diet discussions were initiated by the veterinarian, it may be that clients might feel less inclined to expressly bring up the subject, particularly if there is ambiguity in the veterinarian’s communication about excess weight and diet.

Interestingly, the visits that appeared to have the most client engagement and buy-in to weight loss regimens were visits where helical communication was utilized. As outlined by Adams and Kurtz (31), effective communication follows a helical model that means that a topic is circled back to and repeatedly touched upon. Sign-posting and reintroduction of the weight topic typically utilized humor as reflective listening tool, which seemed to be an effective and well-received approach by the client. Veterinarians who tried to exhaust a topic by approaching it singularly often seemed to encounter greater resistance and did not revisit the topic.

### Humor

In this study, we found that when discussing excess weight, clients and veterinarians frequently utilized humor. Humor frequently offered a means to discuss obesity in a lower stress manner. However, humor use also had the possibility of backfiring and negatively influencing encounters. Frequently, humor was used to report potentially sensitive or unfavorable news, to navigate difficult discussions, to diffuse tension, and to build affiliation around weight and diet discussions. When a client was resistant or normalized weight status, an explanation by the veterinarian regarding obesity management utilizing humor seemed to diffuse tension on delivery and build the relationship between the veterinarian and client. By cushioning the discussion about obesity and building the relationship, humor helped to lay the foundation for a more detailed discussion regarding weight management recommendations.

In human medicine, it is generally thought that the judicious use of humor can facilitate communication, develop the therapeutic relationship, promote bonding, and enhance patient satisfaction (44, 45). Humor has an empathetic quality and may act as a bridge between physician and patient (46), and the same may be true in veterinary medicine. Empathy has been suggested to enhance health care outcomes (31). By fostering a stronger veterinarian–client relationship, empathetic humor appeared to be a vehicle that acknowledged a sensitive topic in a less confrontational manner and seemed to facilitate weight loss explanation, future planning, and treatment of the patient’s excess weight. Humor can assist in encouraging physical comfort and can be a useful coping mechanism in adverse circumstances in human medicine (44). Our findings suggest that humor is used to reframe and diffuse a problem by shifting into a different viewpoint. Humor might also enable clients to distance themselves from issues that can be troublesome or sensitive, such as weight and diet discussions about their cats. While this may appear as a camouflage or detachment from the problem, Tremayne (44) found that physician-initiated humor acted as an agent to address problems obliquely, encouraging sensitive subject matter to be shared. In teaching and learning environments, humor may be used to increase attention, motivate students, and facilitate creative thinking (47). Amid the veterinarian–client relationship, use of humor appeared to increase discussion of weight management, however the impact on client buy-in and adherence to weight loss recommendations is unknown in our study. We propose that sensitive and careful use of humor can be used as a relationship building tool and be helpful for discussing complicated topics such as obesity.

### Patient-Directed Speak

We suggest that patient-directed talk is another modality in which the sensitivity of subject matter plays a role. Veterinarians or clients speak directly to the patients who are unable to respond. As such, patient-directed speak is for the benefit of the overhearing audience rather than the patient itself (48). If the patient is the direct recipient of the delivery of potentially sensitive information that might be critical of the client’s care, the buffered remarks may be heard without requiring a reaction (48). Our findings align with those of Roberts (48) as patient-directed speak is a frequent theme that emerged when both handling the patient and discussing weight topics that might need further discussion. MacMartin et al. (49) reported on the use of “I know” statements highlight that patient-speak also makes the patient a participant of the exam. By verbalizing toward the patient and not directly implicating the client, it created a communication bridge between the client and veterinarian and took the impetus off the client to respond. This may facilitate an increase in response by the client when problem-solving weight management options for the client later on.

### General Discussion

In this study, a lack of clear recommendations furthered communicative misalignment between veterinarian and client and resulted in missed opportunities during the veterinary visits. While we found, in this study, that veterinarians more frequently assessed nutrition during a routine wellness visit rather than a problem visit, AAHA guidelines on dog and cat weight management (15) suggests that veterinarians are responsible for further exploring a patient’s weight and nutrition beyond simply reporting the weight and identifying the patient’s primary diet at every visit. While the weight topic was often initiated by veterinarians in this sample, many discussions fell short of clear assessment, strategies, or identification of the client’s perspective. Vague directions such as advising to “switch to a light food,” or to “cut back” are not effective and can confuse clients (50).

Stivers (36) identified pre-diagnostic commentary as a means of introducing topics and ideas during the human health assessment as a less-formalized means of preceding a formalized diagnosis. Pre-diagnostic commentary can be a vehicle to foreshadow a diagnosis or negotiate a client’s willingness to proceed with a recommendation. Within our data set, pre-diagnostic commentary was often well developed in terms of introducing the weight topic, although often not developed through to a clear assessment, recommendation, and treatment plan. Adams and Kurtz (31) suggested that veterinarians sharing their thinking out loud, explaining and planning within the client–patient dyad, might offer avenues to align client and veterinarian perspectives. With clearer assessments and recommendations from practitioners, improved client adherence and patient body condition may result. Furthermore, the gender distribution of our participants might have an impact on the nature of communications (veterinarians = 75% female; clients = 72% female). In an analysis of veterinarian–client communication during consultations, Shaw et al. (51) showed that female veterinarian–client dyads conduct more relationship-centered appointments, provided more statements that built rapport, and lifestyle-social information was more likely to be provided to female veterinarians from clients.

In our study, exercise and activity were infrequently mentioned within the weight and diet discussions. The primary foci of the veterinarians’ discussions were caloric intake. Less focus on exercise and environment may parallel the lack of literature regarding feline weight loss and its relationship with activity. A study of Western Australian cats found that as cats were bound to a predominantly indoor existence, an increase in obesity rates followed (52). The same author also reported that outdoor cats had a significantly lower body condition than indoor cats, and neuter status, breed, gender, number of cats in a household as well as time spent outside were significantly related to weight status (52). Our data demonstrate the scarcity of investigation into the lifestyle of each patient. This might yield further opportunities to enable the therapeutic relationship and individualize a patient’s weight management plan.

Current models of obesity treatment are without consideration of shared lifestyle, external messaging, and social determinants (53, 54), which is reflected in our results. Within the same archive that our data were obtained, MacMartin et al. (17) showed that veterinarians typically restricted their queries to singular form, “what” prefaced questions such as “what kind of food is she on?” or “what do you feed him?” Such singular form questions may narrow the resulting discussion and eliminate possible topics, such as supplementary foods, treats, table scraps, mousing, and so forth. MacMartin et al. (17) also found that 89% (*n* = 57) of their total sample of clients only reported one or two food items and veterinarians treated these responses as adequate answers, as the majority of veterinarians did not pursue any information about further food items the animal consumes.

Pet obesity is a complicated issue. Our findings illustrate the confounding factors that clients experience in managing obesity of their cats. Perceived obstacles, such as feeding in multiple cat homes, measuring intake and individual food preference, multiple family members, and their buy-in, different feeding habits, are some of the challenges mentioned by clients in this data set. Jyrinki and Leipämaa-Leskinen (55) suggested that pets are seen by people as an extension of self. As such, there are powerful biological and social forces that influence one’s relationship with their pet and the client response to the assessment and recommendations that can further complicate the situation. Another confounding factor is asymmetry between veterinary assessments, where different veterinarians within the same practice have different opinions about a patient’s obesity status or management priorities, which may lead to confusion, lack of clarity, and trust on behalf of the client, such as in the example where the veterinarian undermined his colleague’s previous assessment. By doing so, he also undermined the changes that client had implemented, which might have added to the tension and resistance during the visit.

There is a need for a dynamic and individualized response to obesity management in veterinary medicine. We suggest that obesity is sensitive subject matter to address with clients. Kurtz and Adams (13) outlined the need for a demonstration of greater intentionality from veterinarians when addressing issues of sensitivity. Jones et al. (56) identified pediatric weight management as a sensitive topic to address within the pediatrician–parent–child triad. A broader, one-health approach might lend further insights into the social and environmental determinants that play into human and pet obesity (57). This would align with the needs we have identified in our sample regarding the limited nature of obesity discussions. Furthermore, and as suggested by Degeling et al. (58), treatment plans must take both patients and pet owners into consideration given the links between human health and animal health. Human health understanding can impact pet health, and veterinarians need to take these considerations into account when communicating with pet owners regarding their pet’s health. As such, relationship-centered care practices may allow for veterinarians to address these complex issues with clients by building an understanding of their lifestyle, environment, and bio-social history.

The culture around feeding and diet likely made these discussions more challenging for both the practitioner and client. Pet owners have their own beliefs, perceptions, and values regarding weight and diet that will impact how the interaction between the client and veterinarian unfolds. A client’s food choice often mirrors what they choose for pets (59), such as clients selecting organic food for their cats. Kienzle and Bergler (53) reported that owners of overweight cats were more likely to treat with food items. Colliard et al. (60) found that 59% of cat owners gave extra foods in addition to their daily ration. Consider the previous example of the client who gave their overweight cat treats beyond what the veterinarian had provisioned, despite the veterinarian’s recommendation to only give a few. This might indicate treating pets are important to a client. It is possible that a prescribed food change from a veterinarian might create a better obesity management strategy from the veterinarian’s perspective, but the client might resist because it is not congruent with their values (55). In addition, curbing treating behaviors or reducing feeding portions might be resisted if these practices are seen as excluding the pet from normal family life (55). Kurtz and Adams (13) suggested that when crossing culture between veterinarian and pet owner/client, the use of process skill sets that facilitate sensitive communication are important. Failure to understand the client’s culture of home environment and point of view may lead to communication misalignment. We suggest that increased intentionality toward an exploration of client perspective, open-ended questioning and clear explanation and planning yields a more aligned discussion between veterinarian and clients around obesity and weight management.

### Limitations

We note that results of this type of qualitative analysis are difficult to generalize to different circumstances or individuals. Rather, readers of this study are reminded that before generalizing the results of this study, that they might first consider the degree to which their circumstances mirror those of this study. Findings from this study can be used to reflect upon our own perceptions and expectations of obesity and diet communications. These visits were recorded in 2006 and, therefore, may not reflect the current and dynamic knowledge in veterinary curricula and practice. Dental-focused diets were especially promoted by veterinary nutrition companies and veterinarians during the time these data were collected. Since the time these data were collected, client education as a critical component for obesity prevention is becoming recognized (61).

It is not known in this study how in-depth weight and diet management discussions impact duration of the consultations, although Shaw et al. (61) demonstrated that consultations that adopt a biolifestyle-social communication pattern are significantly shorter than those that focus on biomedical (62) and may be an avenue for future research. These data were counted in one region of one province in Canada, and socioeconomic and demographic factors of that region might influence the nature of these data. As with other studies, controlling for bias is an important consideration. Whereas salient themes—communicative misalignment between veterinarian and client, humor, resistance, speaking to/for the patient, and oblique or absent messaging—emerged from these visits, almost half of our data comes from only a few veterinarians. Also, future studies might investigate weight and diet outcomes in relation to these discussions.

## Conclusion

The overall purpose of this paper was to explore and develop a deeper understanding of obesity and diet communications in veterinary practice between veterinarians and cat-owning clients. The data that emerge from these recorded visits are the participants’ own words within the context of the veterinary visit, and when subjected to qualitative analytical techniques provided the researchers with an appreciation of the nature of obesity and diet discussions in practice and reveal themes that emerge in these discussions. We believe that analysis of real-life interactions can add to a baseline for how veterinarians are communicating with clients regarding obesity and diet and identify further avenues for research and development. While this data set is very rich, the relatively low incidence of obesity discussions in our sample is reflective of this cross-sectional data set.

The insights gained in this study, although not definitive, might benefit the veterinary community by providing context and insight into how multi-faceted weight management is for cat owners and as an important starting point for veterinarians to consider their own practices regarding sensitive topic discussions. Future research about obesity management communications for veterinarians can further disentangle some of its complexities. By investing in this effort, and further developing curriculum to veterinarians and other allied health members to provide clear assessment, explanation and planning, and treatment follow up might help manage feline obesity communication asymmetries. This might allow veterinarians to consistently provide satisfying obesity and diet discussions with clients and to understand their individual viewpoints.

## Ethics Statement

This study was carried out in accordance with the recommendations of University of Calgary Conjoint Faculties Research Ethics Board and University of Guelph Natural, Physical and Engineering Sciences Review Board, with written informed consent from all subjects. All subjects gave written informed consent in accordance with the Declaration of Helsinki. The protocol was approved by the University of Calgary Conjoint Faculties Research Ethics Board, study ID number REB03-055 and University of Guelph Natural, Physical, and Engineering Sciences Review Board, study ID number 09JN026.

## Author Contributions

AP is the corresponding author and did the research and writing of the article as part of a Master’s thesis. CA was AP’s graduate supervisor and was involved extensively throughout all phases of the research and writing of this article. CA was the original principal investigator during the data collection in 2006 and supervisor to JC. JC and MR were graduate committee members and provided feedback in developing the research proposal, critiqued aspects of data analysis and supported in revisions of the paper. All authors have given approval for the article submitted for publication.

## Conflict of Interest Statement

The authors declare that the research was conducted in absence of any commercial, financial, or other relationships that might be interpreted as representing a potential conflict of interest.
